# Impact of Reirradiation Utilizing Fractionated Stereotactic Radiotherapy for Recurrent Glioblastoma

**DOI:** 10.7759/cureus.53001

**Published:** 2024-01-26

**Authors:** Lisa B Shields, Patrick O'Dell, Michael W Daniels, Parag R Sevak, Hilary A Highfield, Kaylyn D Sinicrope, David A Sun, Aaron C Spalding

**Affiliations:** 1 Norton Neuroscience Institute, Norton Healthcare, Louisville, USA; 2 Norton Cancer Institute, Norton Healthcare, Louisville, USA; 3 Bioinformatics and Biostatistics, University of Louisville, Louisville, USA; 4 Clinical Pathology Accreditation (CPA) Laboratory, Norton Healthcare, Louisville, USA

**Keywords:** stereotactic radiation, recurrence, glioblastoma, radiation oncology, neuro-oncology

## Abstract

Background: Patients with recurrent glioblastoma (GBM) have limited treatment options. This study determined whether patients with recurrent GBM treated with initial radiation/temozolomide (TMZ) and reirradiation using fractionated stereotactic radiotherapy (FSRT) had improved outcomes.

Materials and methods: We identified 95 patients with recurrent GBM, 50 of whom underwent FSRT at recurrence and 45 who had systemic treatment only (control). The median total FSRT dose at the time of GBM recurrence was 30 Gy in five fractions of the gadolinium-enhanced tumor only.

Results: With a median follow-up of 18 months, the progression-free survival (PFS) and overall survival (OS) following initial GBM diagnosis were longer in the reirradiation group compared to the control group (13.5 vs. 7.5 months [p=0.001] and 24.6 vs. 12.6 months [p<0.001], respectively). For patients who underwent reirradiation, the median time interval between the end of the initial radiation and reirradiation was 15.2 months. The median OS after GBM recurrence was longer in the reirradiation group versus the control group (9.9 vs. 3.5 months [p<0.001]), with a one-year OS survival rate of 22%. The hazard ratio for death of patients in the reirradiation group was 0.31 [0.19-0.50]. The reirradiation group had a higher percentage of patients who received bevacizumab (BEV, 62.0% vs. 28.9%, p=0.002) and a lower percentage of patients whose TMZ was discontinued due to toxicity (8.0% vs. 28.9%, p=0.017) compared to the control group.

Conclusions: Reirradiation utilizing FSRT was associated with improved PFS and OS after GBM recurrence compared to the control group who did not receive additional irradiation.

## Introduction

Glioblastomas (GBMs) are the most common and aggressive primary brain tumors in adults, with an incidence of 3 per 100,000 in the United States [1-3]. GBMs represent 54% of all gliomas and 16% of all intracranial tumors (primary and metastatic) [4]. Management of GBM at initial diagnosis is well-established, consisting of maximum safe resection, localized radiotherapy in conjunction with the oral alkylating agent temazolomide (TMZ), followed by adjuvant TMZ [3-7]. TMZ interferes with DNA synthesis by causing cross-linkage between strands and DNA breakage, which prevents tumor cell division [8]. Despite aggressive treatment, the median progression-free survival (PFS) remains seven to nine months, the overall survival (OS) is 12-15 months, and the five-year survival rate is <5% [3,4,9,10]. Two specific prognostic factors are associated with better survival: specifically, the isocitrate dehydrogenase (IDH)1 mutation and the methylation status of the methyl-guanine methyl transferase gene (MGMT) promoter. Patients with an IDH1-mutant glioblastoma reportedly have a better OS than patients with IDH1-wild-type anaplastic astrocytomas, suggesting that IDH status is more prognostic than histologic grade [11]. Additionally, low levels of MGMT methylation in tumor tissue are associated with longer survival in patients with GBM [3]. Methylation of the MGMT promoter region leads to epigenetic silencing of the MGMT gene, which confers sensitivity to alkylating chemotherapeutic agents such as TMZ [2]. The median OS for patients whose tumor has MGMT promoter methylation is 18.2 months, compared to 12.2 months for patients whose tumor does not [12]. Another form of treatment is Optune®, which provides specific tumor-treating fields and disrupts mitosis, which is associated with improvements in OS and 2, 3, and 4-year survival rates [13,14].

At GBM recurrence, a well-defined standard of care is lacking. The median PFS is 10 weeks at recurrence, with a median OS of 30 weeks [2]. Most recurrences occur within or just outside the previously irradiated brain [5,15]. Patients may select repeat surgery, reirradiation, systemic therapy, or supportive care [10,16]. Although only one-fourth of patients are considered for repeat surgery at recurrence due to the infiltrative nature of this tumor, those who are younger, have a good performance status, undergo initial surgery more than six months prior to the recurrence, and have a gross total resection (GTR) or near GTR have survival benefits (median OS 6-17 months) [2,7,10,17]. Molecular analysis performed during the second surgery may also direct further treatment [8].

While radiation is well-defined for newly diagnosed GBM, its use for tumor recurrence is unclear. The most desirable radiation dose regimen, target volumes, and stereotactic systems are not standardized for tumor recurrence. Improved outcomes have been reported for younger patients with a good performance status (Karnofsky Performance Scale >60%), smaller tumor size (<40 mm), frontal lobe tumors, progression more than six months from the initial surgery, Eastern Cooperative Oncology Group (ECOG) performance status 0, 1, or 2, and those who have a longer duration between the initial radiation and reirradiation [5,6,15,17]. Reirradiation may consist of a single high-fraction dose for small tumor volumes or hypofractionated radiotherapy, where the total dose is divided into several fractions for large tumor volumes [8]. Stereotactic radiotherapy delivers high targeted radiotherapy doses to the tumor while sparing the surrounding normal brain tissue [8]. While reirradiation may be promising, with a reported median OS between 6 and 12 months, it remains controversial due to the risk of toxicity, including radionecrosis [5,9,10].

Single-agent or combination chemotherapy may be appropriate for patients with recurrent GBM. TMZ may be beneficial for recurrence, especially in patients who had a favorable response to initial TMZ treatment. Approved by the US Food and Drug Administration in 2009 for recurrent GBM, bevacizumab (BEV) has response rates of 30%, with six-month PFS rates between 18% and 42% and a median OS duration between 6.5 and 9.2 months [7,10,18]. BEV, a humanized monoclonal antibody against vascular endothelial growth factor (VEGF), binds and inhibits VEGF, interfering with the tumor blood supply and preventing vessel proliferation [8]. Additional treatments at recurrence include the chemotherapy PCV (procarbazine, lomustine, and vincristine) or other single-agent nitrosoureas, specifically, DNA alkylating agents with high lipophilicity that permit blood-brain barrier penetration [7,8]. In Marwah et al.’s systematic review and meta-analysis of 2084 patients who were treated with reirradiation versus systemic therapy versus combination therapy for recurrent high-grade glioma, combination therapy may improve OS and PFS with acceptable toxicities in patients with recurrent high-grade glioma [19]. Additionally, combining BEV with reirradiation prophylactically reduces radionecrosis. We report 95 patients with recurrent GBM, 50 of whom underwent reirradiation using fractionated stereotactic radiotherapy (FSRT) for a recurrence, and 45 who had systemic treatment only. The clinical characteristics and survival analysis of patients with recurrent GBM are presented. The benefits of treating patients with FSRT at GBM recurrence are also discussed.

## Materials and methods

Under an Institutional Review Board (IRB)-approved protocol and according to the Declaration of Helsinki, we performed an 11-year (November 1, 2013 to October 31, 2022) retrospective review of consecutively treated patients with recurrent GBM. All patients underwent fractionated irradiation as per standard guidelines at GBM diagnosis following either biopsy, GTR, or subtotal resection (STR). Patients underwent CT simulation with 1 mm axial slice imaging fused with both the gadolinium-enhanced T1 and the T2 FLAIR MRI brain images acquired within a week of simulation. The majority of patients were treated with a simultaneous integrated boost technique with 30 identical fractions. The low dose volume encompassing the T2 FLAIR, gadolinium-enhanced T1, and residual tumor or tumor bed received 180 cGy daily for a total dose of 5400 cGy. The high dose target was defined as the gadolinium-enhanced T1 images combined with either the tumor bed for patients who underwent a GTR or with the residual tumor for other patients; this target received 2 Gy per fraction for a total dose of 60 Gy. Patients were analyzed with the intention of being treated, as five patients were not able to complete their initial course of irradiation due to a change in medical status. All patients also received concurrent TMZs.

All patients were followed in the neuro-oncology program no less than every four months. Patients were deemed to have a recurrence date at the time of Response Assessment in Neuro-Oncology (RANO) imaging criteria demonstrating progression or the date of new neurologic symptoms. At the time of recurrence, all patients were presented to the neuro-oncology tumor board and evaluated by neurosurgery, neuro-oncology, and radiation oncology. Patients were offered treatment options including repeat invasive neurosurgical intervention, systemic therapy, Optune®, re-irradiation with FSRT, or supportive care based on performance status, tumor recurrence location, and medical comorbidities.

Patients who underwent re-irradiation using FSRT underwent repeat CT simulation with 1 mm axial images fused with a post-gadolinium T1 brain MRI to generate a single target without treating T2 FLAIR abnormalities. SFRT was delivered with 1, 3, or 5 fractions. The median total radiation dose at GBM recurrence was 30 Gy in 5 fractions (Table 1).

**Table 1:** Prescription number of fractions and prescription total dose at glioblastoma recurrence

Number of patients	Prescription number of fractions	Prescription total dose (Gy)
6	1	21
2	2	24
38	5	30
3	10	35
1	30	60

**Figure 1:**
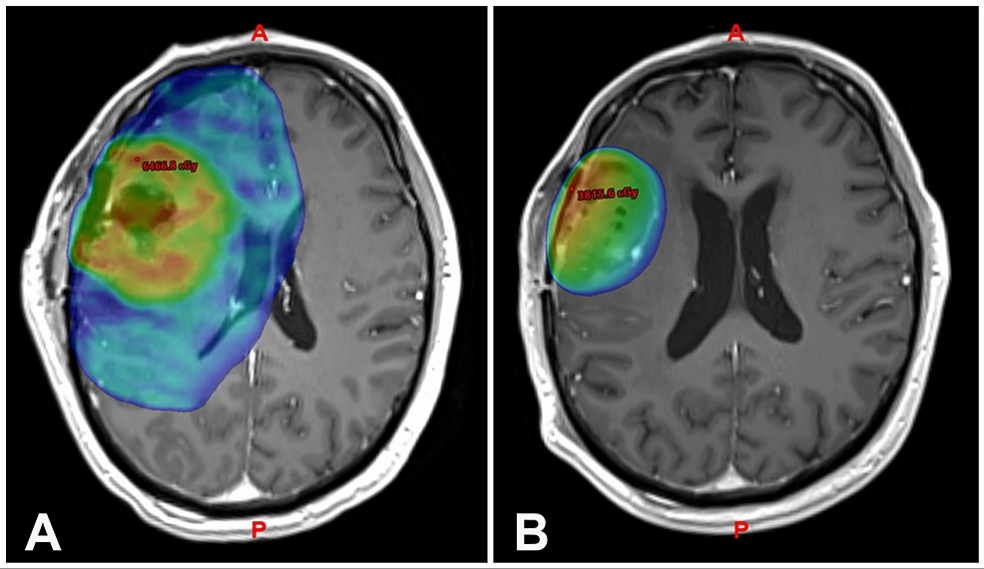
ReFSRT plan demonstrating reduced target volumes on axial MRI T1+1 gadolinium-enhanced sequence (A) Initial target volume for 30 fraction irradiation and (B) ReFSRT target volume

The dose was chosen as there were no toxicities seen from 25 Gy in 5 fractions in other patients with benign brain tumors such as skull-base meningiomas and pituitary adenomas. Since GBM is a malignant histology, we pursued dose escalation. The conformity index was determined for each patient [20]. The reirradiation plan on MRI with Gadolinium contrast highlights the reduced target volumes, from the initial target volume for 30 fraction SRS to the reirradiation target volume (Figure 1).

For the purpose of this study, patients were divided into two groups, specifically: (1) those who underwent FSRT at recurrence and (2) those who had neurosurgical intervention or systemic treatment only (control arm). There were no specific inclusion criteria for FSRT versus standard fraction re-irradiation. Patients who chose supportive care were excluded from this analysis. Data were analyzed to determine the patient’s age, date of initial GBM diagnosis, unifocal vs. multifocal location, MGMT and IDH1 status, tumor laterality, extent of resection at initial diagnosis, treatment with BEV and/or Optune®, radiation volume at the initial diagnosis and at recurrence, ECOG [21] performance status at the initial diagnosis and at recurrence, whether TMZ was discontinued during FSRT due to toxicity, surgical treatment at recurrence, and OS.

Statistical analysis

Nominal and ordinal variables were compared between the reirradiation group and control using chi-squared and t-tests (Wilcoxon rank sum test when normality assumptions were not met), respectively. To evaluate the time to death between the two groups, hazard ratios were calculated using multivariable Cox proportional hazard regressions. Survival curves of the Kaplan-Meier plot were compared using the log-rank test. Spearman correlations were displayed in a correlation matrix, comparing different volumes and times to events. Analysis and diagnostics were performed using R statistical software (v4.2.1, RStudio, Boston, MA), including survival [22], survminer [23], tableone [24], PerformanceAnalytics [25], and ggplot2 [26] packages.

## Results

Clinical characteristics of patients with GBM at initial diagnosis

A total of 95 patients were identified with recurrent GBM, 50 (53%) of whom underwent reirradiation with FSRT at recurrence and 45 (47%) who had systemic treatment only. With a median follow-up of 18 months, at the time of the last follow-up, 82 (86%) of the 95 patients had died. The clinical characteristics of patients with GBM at the initial diagnosis are presented in Table 2.

**Table 2:** Clinical characteristics of patients at the initial glioblastoma diagnosis TMZ: temozolomide, LITT: laser interstitial thermal therapy, GTV: gross total volume, CTV: clinical target volume, PTV: planning target volume

Characteristics	Overall (N=95)	Control (N=45)	Reirradiation (N=50)	P-value
Gender: Male	57 (60.0%)	23 (51.5%)	34 (68.0%)	0.14
Age at diagnosis	58.8 (14.4)	61.2 (12.6)	56.7 (15.2)	0.13
Focality: Unifocal	76 (80.0%)	34 (75.6%)	42 (84.0%)	0.44
MGMT methylation: Yes	30 (32 %)	15 (33%)	15 (30%)	0.85
IDH1: Wild type	74 (78 %)	36 (80 %)	38 (76%)	0.77
Tumor laterality	-	-	-	0.66
Bilateral	7 (7.4%)	3 (6.7%)	4 (8.0%)	
Left	42 (44.2%)	18 (40.0%)	24 (48.0%)	
Right	46 (48.4%)	24 (53.3%)	22 (44.0%)	
Cerebral location	-	-	-	-
Frontal	39 (41.1%)	19 (42.2%)	20 (40.0%)	0.99
Temporal	30 (31.6%)	16 (35.6%)	14 (28.0%)	0.57
Parietal	20 (21.1%)	9 (20.0%)	11 (22.0%)	1.00
Occipital	13 (13.7%)	7 (15.6%)	6 (12.0%)	0.84
Cerebellum	1 (1.1%)	0 (0.0%)	1 (2.0%)	1.00
Brainstem/thalamus/basal ganglia	9 (9.5%)	5 (11.1%)	4 (8.0%)	0.87
Extent of resection	-	-	-	0.85
Biopsy	22 (23.2%)	10 (22.2%)	12 (24.0%)	
Gross total resection	44 (46.3%)	20 (44.4%)	24 (48.0%)	
Subtotal resection	29 (30.5%)	15 (33.3%)	14 (28.0%)	
LITT during surgery: Yes	6 (6.3%)	5 (11.1%)	1 (2.0%)	0.16
Dexamethasone during initial radiation: Yes	50 (52.6%)	25 (55.6%)	25 (50.0%)	0.74
Bevacizumab concurrent with initial radiation: Yes	5 (5.3%)	2 (4.4%)	3 (6.0%)	1.00
Bevacizumab at all: Yes	44 (46.3%)	13 (28.9%)	31 (62.0%)	0.002
Optune® initially prescribed: Yes	2 (2.1%)	1 (2.2%)	1 (2.0%)	1.00
Optune® ever during their course of treatment: Yes	31 (32.6%)	14 (31.1%)	17 (34.0%)	0.94
Initial radiation volumes (cm^3^)	-	-	-	-
GTV6000	38.2 (40.1)	44.0 (48.9)	31.5 (26.2)	0.16
CTV6000	128.5 (120.8)	151.9 (151.8)	101.3 (61.1)	0.06
PTV6000	156.5 (94.9)	171.1 (107.3)	141.9 (79.1)	0.16
GTV5400	69.9 (188.5)	76.6 (185.3)	62.2 (194.6)	0.75
CTV5400	265.2 (241.2)	273.5 (235.8)	255.9 (250.1)	0.76
PTV5400	337.9 (258.0)	349.7 (258.2)	325.9 (260.7)	0.69
ECOG performance status	-	-	-	0.17
0	11 (11.6%)	8 (17.8%)	3 (6.0%)	
1	66 (69.5%)	28 (62.2%)	38 (76.0%)	
≥2	18 (18.9%)	9 (20.0%)	9 (18.0%)	
Number of radiation treatments at diagnosis	28.9 (4.0)	28.6 (4.6)	29.3 (3.4)	0.42
TMZ discontinued due to toxicity: Yes	17 (17.9%)	13 (28.9%)	4 (8.0%)	0.02

At the initial GBM diagnosis, only 30 (32%) of the 95 patients had MGMT methylation, while 74 (78%) of the 95 patients had wild-type IDH1 expression, meaning that this cohort of patients overall had negative pathologic prognostic markers. Similarly, less than half (44 of the 95 patients) were able to undergo GTR. Seventy-seven (81%) of the 95 patients had an ECOG of 0 or 1, indicating the majority of patients had minimal limitations due to their diagnosis despite the pathologic markers and the extent of the residual GBM after neurosurgical intervention. Although there was no way to know at the time of initial diagnosis, Table 2 indicates that between the patients who subsequently chose or declined FSRT at recurrence, there was not an imbalance between known prognostic factors that could confound the impact of recurrent treatment arms. Specifically, MGMT methylation, IDH1 status, and rate of GTR did not differ between the control and reirradiation groups (33 vs. 30% [p=0.85]; 80% vs. 76% [p=0.77]; and 44% vs. 48% [p=0.85], respectively). The reirradiation group had a higher percentage of patients who received BEV (62.0% vs. 28.9%, p=0.002) and a lower percentage of patients whose TMZ was discontinued due to toxicity (8.0% vs. 28.9%, p=0.017) compared to the control group. The remaining known clinical factors did not differ between the groups and are listed in Table 2. There were minimal immediate toxicities observed, which consisted mainly of transient fatigue and cranial alopecia. Radiation necrosis was not observed in this cohort. 

Clinical characteristics of patients with GBM at recurrence

The clinical characteristics of patients with GBM at recurrence are depicted in Table 3.

**Table 3:** Clinical characteristics of patients at glioblastoma recurrence PFS: progression-free survival, OS: overall survival, LITT: laser interstitial thermal therapy

Characteristics	Overall (N=95)	Control (N=45)	Reirradiation (N=50)	P-value
Age	59.9 (13.9)	62.0 (12.5)	58.1 (15.0)	0.178
PFS following initial glioblastoma diagnosis (months)	10.2 [6.0, 15.5]	7.5 [4.2, 11.9]	13.5 [7.9, 19.7]	0.001
OS following initial glioblastoma diagnosis (months)	17.5 [12.4, 27.9]	12.6 [8.6, 16.8]	24.6 [17.7, 36.0]	<0.001
PFS from the end of initial radiation	7.7 [3.5, 13.1]	5.1 [2.0, 8.4]	10.8 [5.4, 17.8]	0.001
OS from the end of initial radiation (months)	6.8 [3.5, 11.5]	3.5 [2.3, 5.3]	9.9 [7.4, 18.4]	<0.001
Median duration between end of initial radiation and reirradiation (months)	15.2 [9.9, 23.5]	-	15.2 [9.9, 23.5]	-
Median duration between end of reirradiation and last contact/death (months)	6.0 [3.7, 11.7]	-	6.0 [3.7, 11.7]	-
ECOG performance status	-	-	-	0.205
0	4 (4.2%)	1 (2.2%)	3 (6.0%)	
1	32 (33.7%)	12 (26.7%)	20 (40.0%)	
≥2	59 (62.1%)	32 (71.1%)	27 (54.0%)	
Field at recurrence	-	-	-	0.923
In field	70 (73.7%)	34 (75.6%)	36 (72.0%)	
Out of field	18 (18.9%)	8 (17.8%)	10 (20.0%)	
Field edge	7 (7.4%)	3 (6.7%)	4 (8.0%)	
Focality: Unifocal	64 (67.4%)	33 (73.%)	31 (62.0%)	0.338
Tumor laterality	-	-	-	0.140
Bilateral	9 (9.5%)	3 (6.7%)	6 (12.0%)	
Left	37 (38.9%)	14 (31.1%)	23 (46.0%)	
Right	49 (51.6%)	28 (62.2%)	21 (42.0%)	
Location	-	-	-	-
Frontal	35 (36.8%)	16 (35.6%)	19 (38.0%)	0.973
Temporal	32 (33.7%)	17 (37.8%)	15 (30.0%)	0.560
Parietal	22 (23.2%)	6 (13.3%)	16 (32.0%)	0.056
Occipital	11 (11.6%)	8 (17.8%)	3 (6.0%)	0.141
Cerebellum	1 (1.1%)	1 (2.2%)	0 (0.0%)	0.958
Brainstem/thalamus/basal ganglia	10 (10.5%)	5 (11.1%)	5 (10.0%)	1.00
Extent of resection	-	-	-	0.074
Biopsy	19 (20.0%)	7 (15.6%)	12 (24.0%)	
Gross total resection	16 (16.8%)	4 (8.9%)	12 (24.0%)	
Subtotal resection	15 (15.8%)	10 (22.2%)	5 (10.0%)	
No surgery	45 (47.4%)	24 (53.3%)	24 (42.0%)	
LITT during surgery at recurrence: Yes	11 (11.6%)	6 (13.3%)	5 (10.0%)	0.853
Dexamethasone at recurrence	70 (73.7%)	33 (73.3%)	37 (74.0%)	1.00
Optune® at all: Yes	23 (24.2%)	12 (26.7%)	11 (22.0%)	0.772

The total PFS and OS following GBM diagnosis were longer in the reirradiation group compared to the control group (13.5 vs. 7.5 months [p=0.001] and 24.6 vs. 12.6 months [p<0.001]) (Table 3). Furthermore, the duration between the end of the initial radiation and recurrence was greater for patients who underwent reirradiation compared to those who did not (10.8 vs. 5.1, [p=0.001]). The duration between GBM recurrence and last contact or death was longer in the reirradiation versus the control groups (9.9 vs. 3.5 months [p<0.001]).

For the patients who underwent FSRT at recurrence, the median time interval between the end of the initial radiation and reirradiation was 15.2 months [9.9, 23.5]. Furthermore, after completion of reirradiation, the median OS was 6.0 months [3.7, 11.7], with a one-year OS survival rate of 22%.

**Figure 2:**
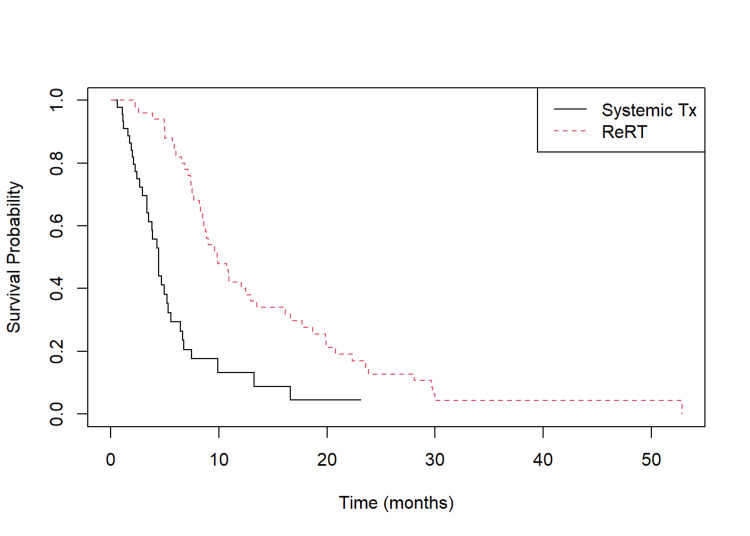
Kaplan-Meier plot of patients who underwent reirradiation at glioblastoma recurrence compared to the control arm Kaplan-Meier plot revealing that patients who underwent reirradiation at glioblastoma recurrence had a longer median OS from the time of recurrence of 9.9 months versus 3.5 months in the control arm (p<0.0001).

As differences between the FSRT patients and those who did not undergo FSRT could confound the interpretation of the impact of reirradiation with FSRT at the time of recurrence, we conducted an analysis of prognostic factors for patients with GBM at the time of recurrence (Table 3). There were no differences in ECOG performance status, the rate of recurrence in the prior high-dose radiation field, the rate of multifocal recurrence, or the location of recurrence. Additionally, there were no differences in interventions at recurrence, with similar rates of extent of resection, laser interstitial thermal therapy (LITT), dexamethasone administration, or Optune® use between the two groups. The time to recurrence and time to death/follow-up were correlated (rs=0.21, p<0.05) (Figure 2).

Survival analysis of patients with recurrent GBM

Patients who underwent reirradiation at GBM recurrence had a longer median OS from the time of recurrence of 9.9 [7.4, 18.4] months versus 3.5 [2.3, 5.3] months in the control arm (p<0.0001) (Table 3, Figure 2). Survival analysis revealed the hazard ratio (HR) for death of the patients in the reirradiation group as 0.31 [0.19-0.50]. In order to identify correlates with outcome, a multivariate analysis was performed (Table 4).

**Table 4:** Multivariate analysis of factors associated with survival after reirradiation using FSRT HR: hazard ratio, ReRT: reirradiation, FSRT: fractionated stereotactic radiotherapy, WT: wild type, ECOG: Eastern Cooperative Oncology Group

Positive Factor	HR	95% CI	P-value
ReRT with FSRT vs No ReRT	0.17	0.08–0.34	<0.001
IDH1 mutation vs WT	0.19	0.05–0.73	0.016
Occipital recurrence vs other lobes	0.29	0.09–0.88	0.029
Negative factor	-	-	-
ECOG of 1 vs 0	2.67	0.98–7.56	0.064
ECOG of 2 vs 0	3.77	1.20–11.81	0.023

When adjusting for location, IDH1, age at diagnosis, and initial ECOG performance, the risk for death was further reduced with FSRT (HRadj=0.17 [0.08, 0.34], p<0.001). Additional variables significantly associated with reduced risk of death were IDH1 mutation (HRadj=0.19 [0.05, 0.73] p=0.016) and occipital location (HRadj=0.29 [0.09, 0.88], p=0.029). We also found that worsening performance status was associated with an increased risk of death after recurrence, with an ECOG score of 1 vs. 0 (HRadj=2.67 [0.98, 7.56], p=0.064) and an ECOG score of 2 vs. 0 (HRadj=3.77 [1.20, 11.81], p=0.023).

## Discussion

The use of radiation for recurrent GBM has greatly expanded with the advent of radiosurgery, which permits improved spatial target localization. Precise, focused delivery of highly effective target doses is attained with normal tissue sparing through steep dose gradients. In a comprehensive review by Minniti et al. of 901 patients between 2005 and 2020 who underwent SRS for recurrent GBM, the median dose was 15-18 Gy for a treated volume between 4 and 10 ml [10]. The PFS ranged from 4.4 to 6 months, and the OS was 7.5-13 months. Gamma knife was the most common SRS modality, although hypofractionated treatments were frequently delivered with Cyberknife and LINAC. These authors reported that increased survival rates were observed with SRS and systemic therapies compared to SRS alone. Minniti et al. also analyzed the use of hypofractionated SRT for recurrent glioblastoma in 18 studies of 976 patients between 2005 and 2020, 17 of which utilized the LINAC SRS modality [10]. Moderate (2.5-3.5 Gy per fraction) or high-dose (5 Gy or more per fraction) hypofractionated treatments were delivered. In 10 of these previous studies with 733 patients using total doses of 30-45 Gy delivered in 2.5-4.0 Gy per fraction, the median OS ranged from 7.5 to 12.5 months. A survival time of 7.3 to 12.5 months was observed in eight studies of 272 patients who received high-dose hypofractionated SRT at doses of 25-35 Gy in 5-7 Gy per fraction. Large retrospective multicenter studies by Combs et al. and Navarria et al. using either SRS or hypofractionated SRT for recurrent GBM revealed a similar median OS duration of 8 and 10 months, respectively [27,28].

The median PFS and OS were 7 and 9 months, respectively, in a prospective analysis by Greenspoon et al. of 31 patients treated with fractionated SRS and TMZ at GBM recurrence [1]. The six-month PFS was 60%. Prescription doses ranged between 25 and 35 Gy, depending on the size of the PTV, and were delivered in 5 fractions. These authors noted a statistically significant improvement in survival in the small (<3 cm) GTV subgroup compared to the large GTV subgroup (median survival 10.5 vs. 8.7 months, p<0.05). In a study by Dincoglan et al. of 28 patients who received hypofractionated SRT using the LINAC (25 Gy in 5 fractions), the median follow-up was 42 months [29]. The median time interval between primary chemoradiotherapy and HFSRT was 11.2 months. The median PFS and OS from reirradiation were 5.8 and 10.3 months, respectively. These authors reported that prognostic factors associated with a longer OS included a longer interval between initial treatment and recurrence, a smaller PTV size, KPS ≥70, and a younger age. Similar to the study by Dincoglan et al., Gigliotti et al. evaluated 25 patients who were re-irradiated with LINAC-based SRS and fractionated SRT at GBM recurrence (median dose of 25 Gy in 5 fractions) [30]. The median OS after the initial diagnosis was 39 months. After salvage treatment with SRS or FSRT, the median OS was nine months, with a one-year OS rate of 29%.

The survival rates in the current study using FSRT at GBM recurrence concur with the studies by Dincoglan et al. and Gigliotti et al. using the same FSRT modality. While the radiation dose was slightly higher in our study (30 Gy in 5 fractions compared to their dose protocols of 25 Gy in 5 fractions), the median OS after reirradiation was similar: six months in the present study compared to 10 and 9 months in Dincoglan et al.’s and Gigliotti et al.’s works, respectively. The survival time between the end of initial radiation and reirradiation was longer in our study compared to that of the study by Dincoglan et al. (15.2 vs. 11.2 months, respectively). The one-year OS rate after reirradiation was similar between our study and the work by Gigliotti et al., specifically, 22% versus 29%, respectively. Unlike the works of Dincoglan et al. and Gigliotti et al., our study highlights a control arm of patients who did not receive irradiation at GBM recurrence. We determined that the patients who underwent FSRT reirradiation had a significantly longer OS at GBM diagnosis and tumor recurrence compared to those who had systemic therapy only at recurrence. Furthermore, patients who underwent reirradiation were more likely to be treated with BEV and were less likely to discontinue TMZ due to toxicity during their initial radiation. Similar to reports in the literature [5,15], the GBM recurrence in our study was in-field in the majority of control patients and those who were treated with salvage radiation.

Strengths and limitations of the current study

The strength of the present study is the large group of patients with recurrent GBM over an 11-year duration who were divided into those who underwent FSRT reirradiation and those who underwent systemic therapy only at recurrence. Through this approach, we were able to effectively evaluate the impact of FSRT reirradiation on tumor recurrence. Our study adds to the burgeoning literature about the significant benefit of reirradiation in the setting of GBM recurrence and highlights the need for more prospective studies that specifically evaluate the use of FSRT reirradiation at tumor recurrence. The limitation of the current study is its retrospective nature and the inherent bias in selecting which patients would undergo reirradiation at GBM recurrence. Additionally, we did not administer validated quality-of-life instruments for patient or family assessment.

## Conclusions

Our study reported 95 patients with recurrent GBM, 50 of whom underwent reirradiation using FSRT at recurrence and 45 who had systemic treatment only. The clinical characteristics and survival analysis of patients with recurrent GBM were presented, as well as the benefits of treating patients with FSRT at GBM recurrence. Our study highlights that in patients diagnosed with recurrent GBM, reirradiation with small-volume stereotactic radiotherapy was associated with a 6 ½ month longer median survival after recurrence compared to a control group who did not receive additional irradiation. Additional analyses are warranted to determine the impact of concurrent systemic therapies with irradiation and underlying tumor patient factors in predicting outcomes.
